# New method for quantification of severity of isolated scaphocephaly linked to intracranial volume

**DOI:** 10.1007/s00381-020-04932-9

**Published:** 2020-10-18

**Authors:** Otto D. M. Kronig, Sophia A. J. Kronig, Léon N. A. Van Adrichem

**Affiliations:** 1 Department of Plastic and Reconstructive Surgery, University Medical Center Utrecht, Utrecht, Netherlands; 2 Department of Plastic and Reconstructive Surgery and Hand Surgery, University Medical Center Utrecht, Heidelberglaan 100, 3584 CX Utrecht, The Netherlands

**Keywords:** Quantification, Cranial suture, Synostosis, Severity, Scaphocephaly

## Abstract

**Purpose:**

The aim is to implement Utrecht Cranial Shape Quantificator (UCSQ) for quantification of severity of scaphocephaly and compare UCSQ with the most used quantification method, cranial index (CI). Additionally, severity is linked to intracranial volume (ICV).

**Methods:**

Sinusoid curves of 21 pre-operative children (age < 2 years) with isolated scaphocephaly were created. Variables of UCSQ (width of skull and maximum occiput and forehead) were combined to determine severity. CI was calculated. Three raters performed visual scoring for clinical severity (rating of 6 items; total score of 12 represents most severe form). Pearson’s correlation test was used for correlation between UCSQ and visual score and between both CIs. ICV was calculated using OsiriX. ICV was compared to normative values and correlated to severity.

**Results:**

Mean UCSQ was 22.00 (2.00–42.00). Mean traditional CI was 66.01 (57.36–78.58), and mean visual score was 9.1 (7–12). Correlations between both traditional CI and CI of UCSQ and overall visual scores were moderate and high (*r* = − 0.59; *p* = 0.005 vs. *r* = − 0.81; *p* < 0.000). Mean ICV was 910 mL (671–1303), and ICV varied from decreased to increased compared to normative values. Negligible correlation was found between ICV and UCSQ (*r* = 0.26; *p* > 0.05) and between ICV and CI and visual score (*r* = − 0.30; *p* > 0.05 and *r* = 0.17; *p* > 0.05, respectively).

**Conclusion:**

Our current advice is to use traditional CI in clinical practice; it is easy to use and minimally invasive. However, UCSQ is more precise and objective and captures whole skull shape. Therefore, UCSQ is preferable for research. Additionally, more severe scaphocephaly does not result in more deviant skull volumes.

## Introduction

Scaphocephaly is the most common form of isolated single-suture synostosis and accounts for 40–60% of the cases of craniosynostosis [1, 2]. Premature closure of the sagittal suture results in an elongated anteroposterior and narrowed transverse dimension of the skull (“boatlike head”) [3–5]. A variable phenotype is seen and depends on the region of synostosis of the sagittal suture: anterior part of the suture (frontal bossing), posterior part of the suture (occipital bulging), or complete fusion (both frontal and occipital bulge) [6, 7].

Craniosynostosis alters skull shape and thereby possibly intracranial volume (ICV). An apparent contradiction has been found in previous studies regarding ICV in patients with scaphocephaly; pre-operative ICV in these patients was found to be increased when compared to mean normal values available through Lichtenberg [8–10]. However, other studies found normal or diminished ICV in these patients [11, 12].

Although in general the clinician can relatively easily establish the diagnosis scaphocephaly, it is more difficult to evaluate the severity and its esthetic improvement following surgery. Scaphocephaly can differ in severity and phenotype, depending on the degree of premature suture fusion [6, 7]. Cranial index (CI) is the most commonly used method for evaluation of cranial deformities in scaphocephaly. However, CI only takes length and width into account, and therefore a major disadvantage of this method is that it does not capture the frontal and occipital malformations or its correction [13].

The aim of the current study is to implement UCSQ (Utrecht Cranial Shape Quantificator) for quantification of scaphocephaly [14]. UCSQ is an outline-based method of classification of skull shape deformities. We use external landmarks (soft tissue landmarks (left portion and left and right exocanthion), visible with the bare eye) to determine a reference plane at 4 cm height on CT scan. Following, an algorithm measures distance and angle from center of mass on the plane to the skull outline, leading to sinusoid curves. The resulting curves are specific and characteristic for scaphocephaly. UCSQ has the advantage of capturing the actual skull shape variation with every 3D diagnostic system capturing the surface of the head. Additionally, since there is still controversy concerning ICV of patients with scaphocephaly, these volumes are measured and correlated to the severity of the skull deformity. UCSQ is compared to the gold standard of quantification, CI.

## Material and methods

### Patients

Children with computed tomography (CT) confirmed nonsyndromic scaphocephaly was eligible for inclusion in the present study. Inclusion criteria were an age of 2 years or younger and a pre-operative status. Additionally, pre-operative 3D CT scans needed to be available and needed to contain the complete skull, including orbits and ears. Patients were diagnosed and operated at the Erasmus Medical Center, Sophia Children’s Hospital Rotterdam.

The study was approved by the local Medical Ethics Review Committee (MEC-2016-467). The study was deemed a retrospective clinical study and did not require formal research ethics approval under the Medical Research Involving Human Subjects Act.

### Radiological studies

As stated before, pre-operative 3D CT scans of the skull were collected. CT scans with a slice thickness of more than 3.00 mm were excluded.

Additionally, conventional skull X-rays (anterior-posterior projection and lateral projection) of the included patients were collected.

### Analysis of curve

**Fig. 1:**
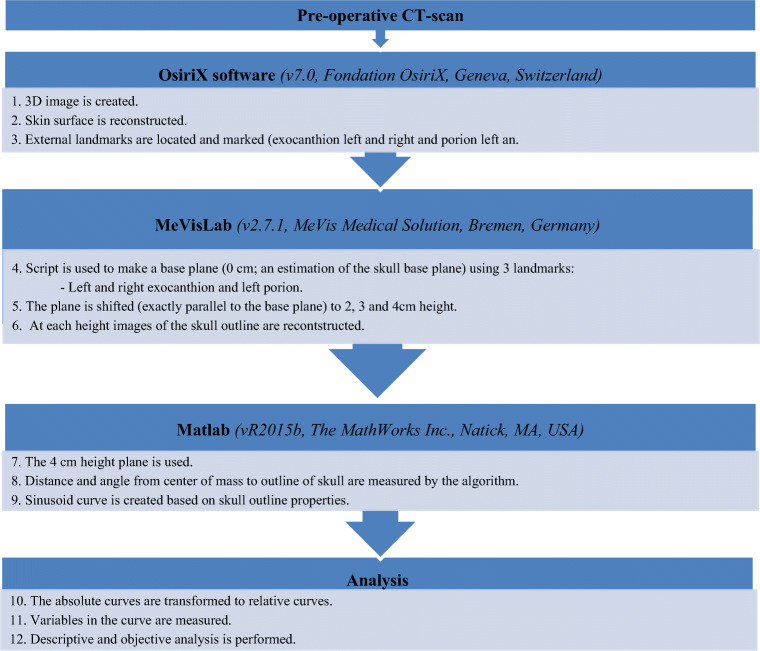
Summary of methods

In this study, we used the UCSQ in order to create sinusoid curves of the included patients (Fig. 1) [14]. The resulting curves were analyzed. Patients with scaphocephaly have a specific and recognizable skull deformity and therefore a specific pattern of the curve. Based on the sinusoid curve, the severity of craniosynostosis can be diagnosed.

**Fig. 2:**
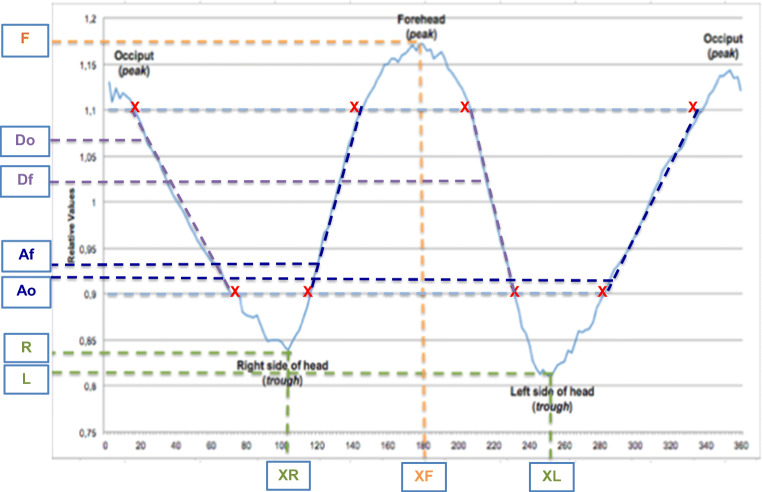
Visualization of the used variables. Abbreviations: Af, abruptness first ascent (forehead); Ao, abruptness second ascent (occiput); Df, abruptness second descent (forehead); Do, abruptness first descent (occiput); F, maximum of forehead; L, minimum value of left side of head; R, minimum value of right side of head; XF, X-value of maximum forehead value; XL, X-value of the minimum value of the width on the left side; XR, X-value of the minimum value of the width on the right side

First, we measured different aspects of the curve. Maximum of forehead (F) and occiput (O), minimum value of the right side of head (trough) (R), and left side (trough) (L) were measured. We calculated the width of the skull (mean of both sides) (R/2 + L/2), difference occiput and sides (O-R/2-L/2), difference forehead and sides (F-R/2-L/2), and difference forehead and occiput (F-O). Additionally, abruptness of the two descents (respectively, Do (descent occiput; first descent) and Df (descent forehead; second descent)) and two ascents (respectively, Ao (ascent occiput; second ascent) and Af (ascent forehead; first ascent)) on the Y-axis were measured between the values of respectively 0.9 and 1.1 (Fig. 2). The described variables (named: variables of the curve) were correlated with the visual score.

Second, the following parameters were combined and used to quantify severity of scaphocephaly according to UCSQ (named: UCSQ): width of the skull and maximum occiput and forehead. Value of width of skull was given a rank number; the narrower the skull, the higher the rank number. Maximum occiput and forehead were added and given rank numbers; the higher these added values, the higher the rank. Both ranking numbers were added.

Third, in order to differentiate between the different levels of severity of scaphocephaly, we used the most distinctive variables of scaphocephaly, namely, forehead, occiput, and the width of the skull. As stated before, the craniosynostosis skull is longer and narrower than the normal skull. Therefore, the difference between the mean values of a control skull for the previous variables and those of a scaphocephaly patient is indicative for severity. We used the mean values from the control patients, as reported in our previous study [15]. The following calculation in order to determine cut-off values and the different classes of severity (mild, moderate, severe) was developed: (Occiput - 1.1) + (Forehead - 1.15) + (1.70 - Width of skull). In this calculation, the values 1.1 and 1.15 are the values for occiput and forehead in control patients. A width of skull lower than 1.70 (2 × 0.85 as value for both sides of the head) is specific for scaphocephaly, and this value will be lower in case of a severe scaphocephaly. Additionally, cut-off values for each subgroup of severity were proposed.

### Visual score

For patients with an available conventional skull X-ray, this X-ray is independently evaluated for width and length of the skull, vertex height, presence of frontal bossing or occipital bulging, and temporal narrowing by three medically trained panel members. When no conventional skull X-ray was available, visual score was performed on CT scan of the skull. The following scoring system (visual score) was assigned for each of the features: score of 0 (normal), 1 (moderate deformity), and 2 (severe deformity). The six parameters were separately evaluated; thus, maximum deformity meant that all the evaluated parameters were severely deformed (a maximum of 12 points could be given). The evaluation is subjective, and no objective criteria are available. Therefore, clinical experience is essential. The normal skull is scored 0 points, the most severe form of scaphocephaly 12 points with presence of a clear frontal bossing and occipital bossing and lower vertex (horse saddle deformity) and increased length (by a fourth) and reduced width (by a fourth) at parietal and temporal level. If 1 of the 6 parameters is between normal and severe, 1 point is scored.

### Cranial index

For patients with an available conventional skull X-ray, CI was measured on this skull X-ray. When unavailable, CI was measured on CT scan of the skull. CI represents the ratio of maximum cranial width to maximum cranial length multiplied by 100 (CI = biparietal diameter (width of skull)/occipitofrontal diameter × 100%). CI gives information of the head shape and the severity of the malformation of the skull.

CI can also be measured using the curve following UCSQ. The maximum cranial width in the new method was measured by multiplying the mean value of both sides of the head by 2, and the maximum cranial length was measured by adding the value of the maximum of forehead to the maximum value of the occiput.

### Calculating the intracranial volume

**Fig. 3:**
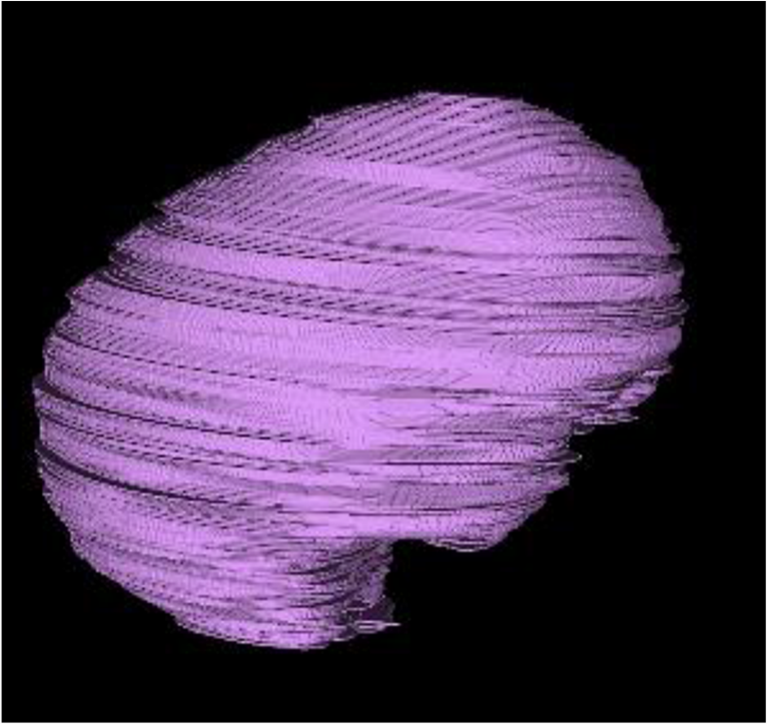
Visualization of calculated and measured ICV

The entire intracranial cavity was considered region of interest (ROI) in order to calculate ICV. CT DICOM images were imported to OsiriX (version 7.0, Fondation OsiriX, Geneva, Switzerland) on Mac OSX. Start slice was considered just above foramen magnum and end slice just beneath vertex of the skull. On each axial slice, the ROI was manually outlined on the inner table of the skull, and defects were manually closed. The total ICV was extrapolated (Fig. 3).

### Lichtenberg’s normal value of intracranial volume

As reference for normal intracranial volumes of (healthy) children, matched for age and sex, we used Lichtenberg’s data, which is the most frequent reference for normal intracranial volume [8].

### Statistical analysis

To assess the level of agreement between observers, intraclass correlation coefficients (ICC) was calculated. The ability of a test to show interobserver reliability was evaluated using the two-way random effects model assuming an average measurement and absolute agreement. An ICC of 1 means perfect reliability, and an ICC of 0 shows poor reliability. ICC for visual score was calculated.

In each patient for each of the evaluated items of the visual assessment, the average of the scores given by the three observers was calculated and used in further statistical evaluation. Each of the calculated averages of the six evaluated items were summed up and resulted in an overall visual assessment, which could range from 0 to 12.

To assess monotonic relationships, between the scored individual features of the visual assessment or the overall visual assessments and each variable of the curve, the Pearson’s correlation coefficient was used. Additionally, UCSQ was correlated to visual score by using Pearson’s correlation coefficient.

Pearson’s correlation between traditional CI and calculated CI of UCSQ, traditional CI, and overall visual score and between calculated CI of the UCSQ and overall visual score were calculated to determine a preferable method of measurement of the CI.

ICV was correlated to both traditional CI and CI of the UCSQ method, visual score, and UCSQ by using Pearson’s correlation coefficient.

The outcomes of the ICC are characterized by Landis and Koch as poor (0.00 to 0.20), fair (0.21 to 0.40), moderate (0.41 to 0.60), good (0.61 to 0.80), or excellent (0.81 to 1.00) [16]. The accepted guidelines for interpreting the correlation coefficients are as follows: + 1 indicates a perfect positive linear relationship, − 1 indicates a perfect negative linear, and 0 indicates no linear relationship. The size of a correlation coefficient can be interpreted as follows: negligible correlation (0.00 to 0.30), low (0.30 to 0.50), moderate (0.50 to 0.70), high (0.70 to 0.90), and very high (0.90 to 1.00) [17].

Statistical analyses were performed using the Statistical Package for the Social Sciences for Windows (Version 21, SPSS Inc., Chicago, IL, USA).

## Results

We included 21 children with nonsyndromic scaphocephaly. Mean age of patients was 7 (2–24) months. There were 18 male and three female patients (85.7% vs. 14.3%). In 14 patients, additional conventional skull X-ray was available.

### Analysis of curve

**Fig. 4:**
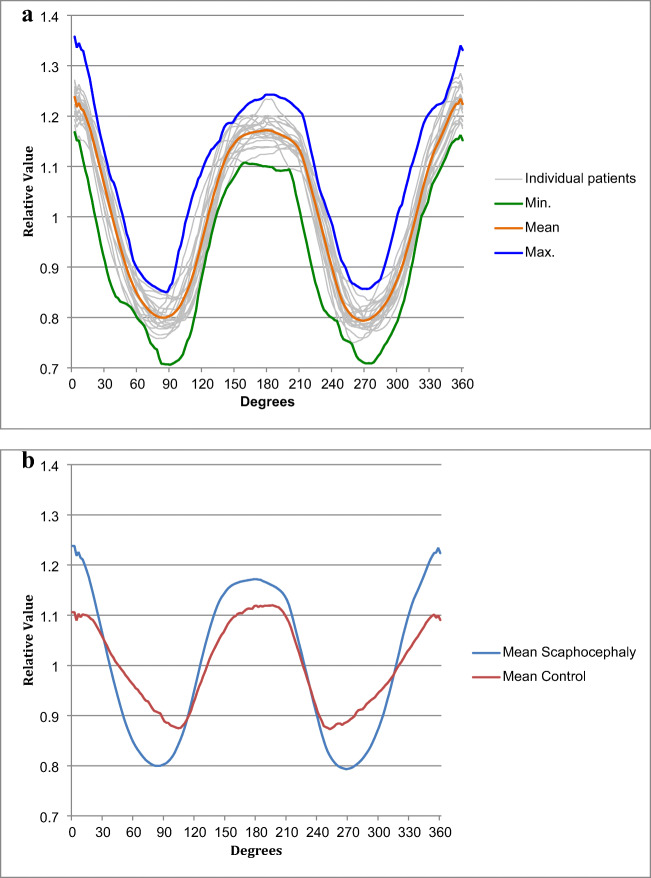
Overview resulting sinusoid curves. **a** Overview graphs of all included scaphocephaly patients and minimal, maximal, and all mean value. **b** Mean curve of included scaphocephaly patients (*N* = 21) and mean curve of control patients (*N* = 5)

Figure 4a shows an overview of the curves of the included patients. Figure 4b shows both the mean curve of scaphocephaly patients and additionally for illustration the mean curve of 5 control patients used in our previous study [15]. Table 1 shows the measured and calculated variables with the use of the generated curves. Mean calculated UCSQ was 22.00 (2.00–42.00).

**Table 1:** Measured and calculated variables of the curves

Variable	Mean (min.–max.)
Maximum of forehead (F)	1.18 (1.10–1.25)
Maximum of occiput (O)	1.24 (1.12–1.30)
Minimum value of right side of head (trough) (R)	0.79 (0.72–0.85)
Minimum value of left side of head (trough) (L)	0.79 (0.72–0.85)
Width of skull (mean of both sides) (R/2 + L/2)	0.79 (0.72–0.85)
Difference occiput and sides (O-R/2-L/2)	0.45 (0.24–0.62)
Difference forehead and sides (F-R/2-L/2)	0.39 (0.25–0.53)
Difference forehead and occiput (F-O)	0.06 (0.01–0.13)
Abruptness descent occiput (Do; first descent)	− 0.51 (− 0.75 to − 0.35)
Abruptness descent forehead (Df; second descent)	− 0.53 (− 0.78 to − 0.26)
Abruptness ascent occiput (Ao; second ascent)	0.52 (0.32–0.78)
Abruptness ascent forehead (Af; first ascent)	0.53 (0.32–0.87)

Mean of the calculation for severity of scaphocephaly ((Occiput - 1.1) + (Forehead - 1.15) + (1.70 - Width of skull)) was 0.30 (0.01–0.60). Mean (Occiput - 1.1) was 0.08 (0.00–0.15), mean (Forehead - 1.15) was 0.09 (0.01–0.18), and mean (1.70 - Width of skull) was 0.12 (0.00–0.27).

### Visual score

**Fig. 5:**
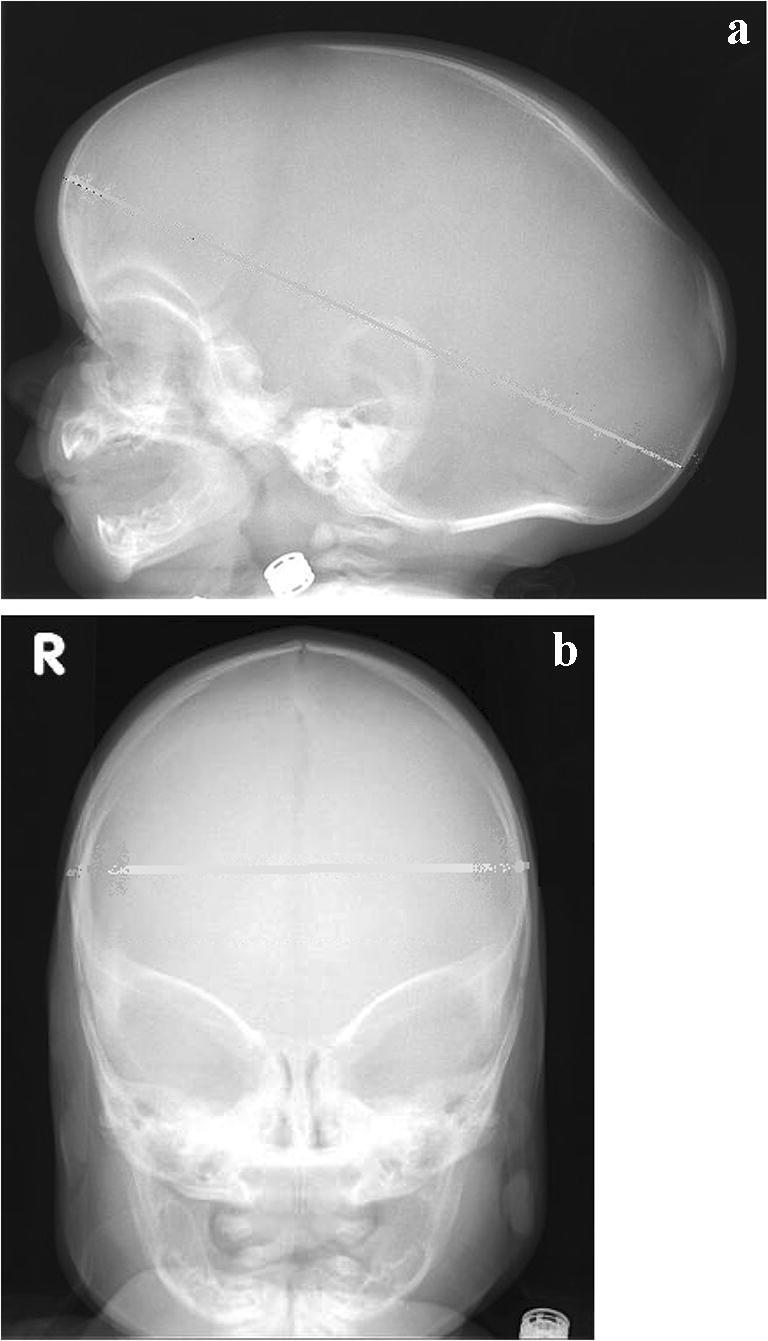
Conventional skull X-ray for visual score. This patient had a visual score of 12. **a** Lateral view. **b** Anterior-posterior view

ICC for visual score was calculated. The level of agreement between the three observers for the values of the width and length of the skull represents perfect reliability (both 0.81; *p* < 0.000), for vertex height and occipital bulging represent good reliability (0.63; *p* < 0.000 and 0.75; *p* < 0.000), and for presence of frontal bossing and temporal narrowing moderate reliability (0.53; *p* = 0.019 and 0.43; *p* = 0.019). For illustration, Fig. 5 shows a patient with the maximum visual score of 12, with presence of a clear frontal bossing and occipital bossing and lower vertex and increased length and reduced width at parietal and temporal level.

Mean visual score was 9.1 (min. 7–max. 12).

#### CI

Mean traditional CI was 66.01 (57.36–78.58), and mean calculated CI of UCSQ was 65.18 (55.41–75.32).

#### ICV

The pre-operative intracranial volumes ranged from 671 mL in a 2-month-old child to an intracranial volume of 1303 mL in a 24-month-old child. Mean intracranial volume of all included patients was 910 mL.

**Fig. 6:**
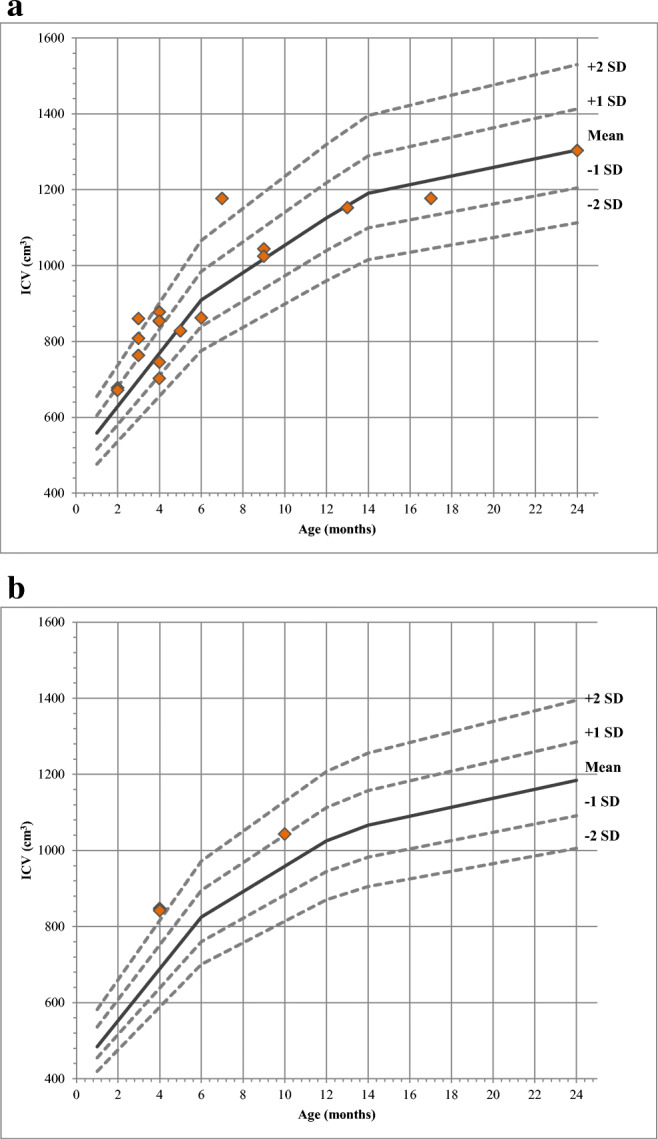
Lichtenberg normative intracranial volume curves are gender- and age-specific curves; ICV measurement of each included patient is plotted on the Lichtenberg normative curves. Orange rhombi indicate patients; dotted lines indicate SD lines (± 1 SD and ± 2 SD) of Lichtenberg mean; continuous line indicates Lichtenberg mean. **a** Boys (*n* = 18). **b** Girls (*n* = 3)

Lichtenberg normative data was used for comparison of normal intracranial volume with pre-operative intracranial volumes of the scaphocephaly patients. Figure 6a and b show measurements of ICV of each included patients plotted in these ranges.

Table 2 shows the ICV of the included patients and the ICV compared to the Lichtenberg’s data. Ten of 18 (56%) boys and 3 of 3 (100%) girls had volumes at or greater than the Lichtenberg mean. In total, 2 boys and 2 girls had an ICV larger than +2 SD. Six of 18 (33%) boys had an ICV lower than the Lichtenberg mean. None of the patients had an ICV lower than – 2 SD.

**Table 2:** Calculated ICV per patient and comparison to Lichtenberg’s data

	Gender	Age *(m)*	ICV *(mL)*	Compared to Lichtenberg’s data
1.	Female	10	1043	+ 1 to + 2 SD
2.	Female	4	848	> + 2 SD
3.	Female	4	842	> + 2 SD
4.	Male	6	862	Mean to − 1 SD
5.	Male	9	1044	Mean to + 1 SD
6.	Male	3	860	> + 2 SD
7.	Male	4	855	+ 1 to + 2 SD
8.	Male	7	1177	> + 2 SD
9.	Male	13	1153	Mean to − 1 SD
10.	Male	3	763	+ 1 to + 2 SD
11.	Male	17	1177	Mean to − 1 SD
12.	Male	2	**677**	Mean to + 1 SD
13.	Male	5	828	Mean to − 1 SD
14.	Male	4	878	+ 1 to + 2 SD
15.	Male	2	671	Mean to + 1 SD
16.	Male	4	853	+ 1 to + 2 SD
17.	Male	4	746	Mean to − 1 SD
18.	Male	9	1025	Mean to + 1 SD
19.	Male	4	702	− 1 to − 2 SD
20.	Male	3	808	+ 1 to + 2 SD
21.	Male	24	1303	At mean

#### Correlations

Overall scores (added value of the evaluated features of conventional skull X-rays) of the visual score show high correlations with *O* (*r* = 0.72; *p* < 0.000), mean width of skull ((R/2 + L/2)) (*r* = − 0.80; *p* < 0.000), and moderate correlation with *F* (*r* = 0.62; *p* = 0.003). Negligible correlation was found for the difference between the forehead and occiput (*r* = 0.12; *p* > 0.05). Overall visual scores show moderate correlation with abruptness of first descent (Do) (*r* = − 0.53; *p* = 0.013), abruptness of second descent (Df) (*r* = − 0.63; *p* = 0.002), abruptness of first ascent (Af) (*r* = 0.61; *p* = 0.003), and abruptness of the second ascent (Ao) (*r* = 0.63; *p* = 0.002).

Additionally, when looking at different items of visual score separately, length of forehead according to visual score was correlated to the extracted value from the curve, showing negligible correlation (*r* = 0.29; *p* > 0.05). Length of occiput according to visual score moderately correlated to extracted value from the curve (*r* = 0.56; *p* = 0.008), and width of skull according to visual score showed moderate correlation to extracted value from the curve (*r* = − 0.66; *p* = 0.001).

Correlation between traditional CI and calculated CI of UCSQ was high (*r* = 0.88; *p* < 0.000).

Correlation between traditional CI and overall visual score show a moderate relationship (*r* = − 0.59; *p* = 0.005). Correlation between calculated CI of UCSQ and overall visual score was high (*r* = − 0.81; *p* < 0.000).

UCSQ was used to quantify the severity based on the selected variables and given ranking numbers. High correlation was found between UCSQ and visual score (*r* = 0.88; *p* < 0.000).

Low and negligible correlation was found between ICV and both traditional CI and CI of UCSQ (*r* = − 0.30; *p* > 0.05 and *r* = − 0.23; *p* > 0.05, respectively). Correlation between ICV and visual score was negligible (*r* = 0.17; *p* > 0.05). Negligible correlation was found between ICV and UCSQ (*r* = 0.26; *p* > 0.05).

## Discussion

The aim of the current study was to evaluate and quantify a group of pre-operative patients diagnosed with isolated scaphocephaly using our UCSQ [15]. Our method is able to differentiate between the different levels of severity in patients with isolated sagittal synostosis.

In addition to CI, several new techniques are introduced in order to classify scaphocephaly; however, these methods are difficult to reproduce and complex [10, 14, 18–22]. No consent has been reached about which method to use in patients with scaphocephaly. The lack of agreement of superiority of any method is partially explained by the insufficiency of available evidence and complexity of the methods.

The first reported and most commonly used method of quantification is the CI, which only takes the length and width of the skull into account, while any other clinical findings or dysmorphology is not. For instance, a cranium with relatively minimal AP length but increased frontal height can be a severe case of scaphocephaly without an appropriately low CI. An updated index of the CI was introduced to differentiate the severity of scaphocephaly, the scaphocephaly severity index (SSI; distance-based approach). In comparison with the CI, the SSI uses calculations on different, perpendicular, transverse planes of CT scans, and additionally, internal or surface landmarks are used [21]. Neither the CI nor the SSI used the actual geometric variation in shape of the skull. A solution for capturing geometric variations of the skull is an outline-based approach [23].

Our UCSQ is a distance- and outline-based approach, which uses recognizable and reproducible surface landmarks and captures the actual geometric variations and cranial width and length of the skull [15]. A major advantage of the outline-based quantification method, instead of the distance-based quantification method, is the ability to represent more aspects of cranial deformation.

In the current study, ICVs were calculated; however, no consistency in pre-operative volume measurements was found. ICV ranged from smaller to bigger than Lichtenberg mean. Four of 21 patients had an ICV larger than + 2 SD, and other ICVs were in the acceptable range. These findings are comparable with earlier reports, noting these differences in volumes [9–12]. Negligible correlation between severity of scaphocephaly according to UCSQ and ICV was found. ICV did not correlate to CI and visual score. These results indicate that the more severe cases of scaphocephaly do not result in more deviant skull volumes, neither smaller nor larger volumes.

The scaphocephalic skull shape is long and narrow, resulting in high values of the maximum occiput and forehead and low values of the width of the skull. Moderate and high correlation is found between overall visual score and forehead and occiput, respectively, indicating a higher score correlates with a longer forehead and occiput. In addition, high correlation was found for width of head, which shows correlation with a higher score and a more narrow side of the head. This indicates that the overall visual score correlates with the individual variables, length, and width; these variables are therefore suited for quantification of severity.

We found that a high correlation was found between UCSQ and visual score, meaning that the deformity visible with the bare eye can be expressed in number by UCSQ. UCSQ can objectively quantify severity of scaphocephaly. Additionally, correlation between UCSQ and visual score was stronger than the correlation between visual score and CI.

Based on our calculation for severity of scaphocephaly ((Occiput - 1.1) + (Forehead - 1.15) + (1.70 - Width of skull)), we propose the following cut-off values in order to classify severity: mild < 0.20, moderate ≥ 0.20 and < 0.35, and severe ≥ 0.35. However, these cut-off values are only based on 21 patients, and further validation of these values is needed in future research.

When looking into separate items of the visual score, length of forehead and occiput according to visual score show less strong correlation to the extracted values from the curve. Correlation between width of skull according to visual score showed moderate correlation to extracted value from the curve. The measurements were higher than visual scores, indicating that the visual scores underestimate the real deformity. The first descent and second ascent in the curves are linked to the occiput; first ascent and second descent are linked to the forehead. However, these measures were not consistently related to the corresponding specific features of the visual score, possibly due to underestimation of the visual score.

We compared traditional CI to the calculated CI using UCSQ. Hereby, a limitation occurs, a possible underestimation of CI by using UCSQ, since it takes the length at 4 cm above the basal plane and not at the level of the largest AP length. The new method (UCSQ) then results in a lower CI compared to traditional CI. However, the outcomes of the traditional CI and the calculated CI of our method showed high correlation. Traditional CI showed moderate correlation with the visual score, and calculated CI of UCSQ showed high correlation; negative correlation was measured in both methods, consistent with our other findings. This implicates that our method outperforms traditional CI. This can be explained by the wide variation in the methodology in determining maximum cranial length and width in practice by using the traditional CI, making CI measurements across different studies difficult to compare. This is improved by UCSQ. However, traditional CI is easy, fast, and applicable in clinical setting and is therefore the method of preference. In the future, the UCSQ will be implemented on 3D photogrammetry to perform curvature analysis. This may result into the advantages of minimal invasive follow-up of the skull growth and gives a good visualization of the morphology of the skull shape; furthermore, this method can provide insight in changes in skull shape due to (varying) surgical techniques in comparison to nonsurgical management.

In this study, we used an objective method for quantification of scaphocephaly, which provides a satisfactory representation of the actual characteristic morphology and can be used for analysis of the outcome of a given treatment. Using a scoring system that reflects the degree of severity would allow researchers to investigate other issues, such as the correlation between the severity of the skull malformation and cognitive outcome for patients with scaphocephaly. Our current advice is to use traditional CI in clinical practice, since it is easy to use and minimally invasive for the patients. However, UCSQ is a promising method of quantification and classification in craniosynostosis patients. Therefore, further research is needed to implement UCSQ in surgical decision-making and skull growth follow-up. Additionally, no strong correlation was found between severity of the skull deformity (visual and according to UCSQ) and ICV; more severe scaphocephaly does not result in more deviant skull volumes.

## Data Availability

Not applicable.

## References

[1] Di Rocco F, Arnaud E, Meyer P, Sainte-Rose C, Renier D (2009). Focus session on the changing “epidemiology” of craniosynostosis (comparing two quinquennia: 1985-1989 and 2003-2007) and its impact on the daily clinical practice: a review from Necker Enfants Malades. Childs Nerv Syst.

[2] Di Rocco F, Arnaud E, Renier D (2009). Evolution in the frequency of nonsyndromic craniosynostosis. J Neurosurg Pediatr.

[3] Bertelsen TI (1958). The premature synostosis of the cranial sutures. Acta Ophthalmol Suppl.

[4] Cohen MM (1988). Craniosynostosis update 1987. Am J Med Genet Suppl.

[5] Virchow R (1851). Über den Kretinismus, namentlich in Franken, und über pathologische Schädelformen. Verh Physikalish Med Ges Wurzburg.

[6] Cohen MM (1993). Sutural biology and the correlates of craniosynostosis. Am J Med Genet.

[7] Vollmer DG, Jane JA, Park TS, Persing JA (1984). Variants of sagittal synostosis: strategies for surgical correction. J Neurosurg.

[8] Lichtenberg R (1960) Radiographic du crane de 226 enfants normaux de la naissance a 8 ans. Impressions digit-formes. Capacite: angles et indices. Thesis, University of Paris

[9] Marsh JL, Jenny A, Galic M, Picker S, Vannier MW (1991). Surgical management of sagittal synostosis. A quantitative evaluation of two techniques. Neurosurg Clin N Am.

[10] Posnick JC, Lin KY, Chen P, Armstrong D (1993). Sagittal synostosis: quantitative assessment of presenting deformity and surgical results based on CT scans. Plast Reconstr Surg.

[11] Fischer S, Maltese G, Tarnow P, Wikberg E, Bernhardt P, Tovetjärn R, Kölby L (2015). Intracranial volume is normal in infants with sagittal synostosis. J Plast Surg Hand Surg.

[12] Lee SS, Duncan CC, Knoll BI, Persing JA (2010). Intracranial compartment volume changes in sagittal craniosynostosis patients: influence of comprehensive cranioplasty. Plast Reconstr Surg.

[13] Maugans TA, McComb JG, Levy ML (1997). Surgical management of sagittal synostosis: a comparative analysis of strip craniectomy and calvarial vault remodeling. Pediatr Neurosurg.

[14] Kaiser G (1988). Sagittal synostosis--its clinical significance and the results of three different methods of craniectomy. Childs Nerv Syst.

[15] Kronig ODM, Kronig SAJ, Vrooman HA, Veenland JF, Jippes M, Boen T, Van Adrichem LNA (2020). Introducing a new method for classifying skull shape abnormalities related to craniosynostosis. Eur J Pediatr.

[16] Landis JR, Koch GG (1977). The measurement of observer agreement for categorical data. Biometrics..

[17] Hinkle DE, Wiersma W, Jurs SG (2003). Applied statistics for the behavioral sciences.

[18] David L, Glazier S, Pyle J, Thompson J, Argenta L (2009). Classification system for sagittal craniosynostosis. J Craniofac Surg.

[19] de Oliveira ME, Hallila H, Ritvanen A, Buchler P, Paulasto M, Hukki J (2011). Feature-invariant image registration method for quantification of surgical outcomes in patients with craniosynostosis: a preliminary study. J Pediatr Surg.

[20] Ratner B (2009). The correlation coefficient: its values range between +1/−1, or do they?. J Target Meas Anal Mark.

[21] Ruiz-Correa S, Sze RW, Starr JR, Lin HT, Speltz ML, Cunningham ML (2006). New scaphocephaly severity indices of sagittal craniosynostosis: a comparative study with cranial index quantifications. Cleft Palate Craniofac J.

[22] Yang S, Shapiro L, Cunningham M, Speltz M, Birgfeld C, Atmosukarto I, Lee SI (2013). Skull retrieval for craniosynostosis using sparse logistic regression models. Med Image Comput Comput Assist Interv.

[23] Ruiz-Correa S, Starr JR, Lin HJ, Kapp-Simon KA, Sze RW, Ellenbogen RG, Speltz ML, Cunningham ML (2008). New severity indices for quantifying single-suture metopic craniosynostosis. Neurosurgery..

